# Study on the thermal stability of nab-paclitaxel during hyperthermic intraperitoneal chemotherapy

**DOI:** 10.1186/s40360-023-00653-2

**Published:** 2023-03-01

**Authors:** Jingjing Zhang, Luya Li, Jintuo Yin, Xidong Zhang, Ying Zheng, Rui Feng

**Affiliations:** Department of Pharmacy, The Fourth Hospital of Hebei Medical University, Shijiazhuang, 050011 P. R. China

**Keywords:** Albumin-bound paclitaxel, Thermal stability, Temperature, Hyperthermic intraperitoneal chemotherapy

## Abstract

**Background:**

Albumin-bound paclitaxel (nab-paclitaxel), as a special targeted preparation of paclitaxel, has the advantages of good curative effect and less side effects in anti-tumor therapy. The existence of the plasma-peritoneal barrier and insufficient blood supply make intravenous drugs hard to reach the peritoneum, while hyperthermic intraperitoneal chemotherapy can solve the difficulty. And compared with systemic medications, HIPEC can also give higher concentrations of chemotherapy drugs in the abdominal cavity, while ensuring lower systemic toxicity. However, at present, there is no relevant report on the clinical study of nab-paclitaxel during intraperitoneal hyperthermic chemotherapy, and its stability under special temperature conditions has not been reported either.

**Methods:**

In this study, We examined three batches of albumin-bound paclitaxel dissolved in saline at different temperatures (25 °C, 37 °C, 41 °C, 42 °C and 43 °C) for the changes of human serum albumin content, human serum albumin polymer content, related substance content, in-vitro release rate, paclitaxel binding rate and paclitaxel content at different temperatures.

**Results:**

Our results demonstrated that the indicators including human serum albumin content, human serum albumin polymer content, in-vitro release rate, paclitaxel binding rate and paclitaxel content were stable to the several temperatures, except that Taxane (0.1%) and other individual impurities in the determination of related substance content fluctuated comparatively widely with the change of temperature. In addition, only Taxane (0.1%) and 7-Epitaxol (1%) were detected.

**Conclusions:**

Overall, albumin-bound paclitaxel is relatively stable to different temperatures (25 °C, 37 °C, 41 °C, 42 °C and 43 °C). This study will lay a foundation for further studies on the albumin-bound paclitaxel during hyperthermic intraperitoneal chemotherapy.

## Introduction

Globally, gastric cancer is the third leading cause of cancer death [1–3]. Approximately 15–50% of patients with advanced gastric cancer have peritoneal carcinomatosis (PC) at diagnosis, and PC was the main cause of post-op recurrence in 35–50% of patients [4, 5]. Hyperthermic intraperitoneal chemotherapy (HIPEC), as a comprehensive therapy involving intraperitoneal perfusion, warming effect, and chemotherapeutic drugs, has been used in primary and secondary peritoneal tumors, as well as an adjuvant treatment for abdominal malignant tumors [6–11].

In recent years, the dosage form of paclitaxel has been increasing continuously, overcoming the shortcomings of traditional paclitaxel injection. Among them, albumin-bound paclitaxel (nab-paclitaxel) is a solvent-free, 130 nm albumin-paclitaxel complex [12, 13]. Compared with traditional paclitaxel, nab-paclitaxel, which does not contain polyethoxylated castor oil, can minimize the risk of hypersensitivity reactions [14, 15]. In addition, since this form does not require hydrated ethanol as a solvent, it can be used for patients with alcohol intolerance. Furthermore, compared to solvent-based paclitaxel, high-dose nab-paclitaxel can be administered in a shorter infusion time. At the same dose, the paclitaxel dose of nab-paclitaxel is 33% higher than that of solvent-based paclitaxel, which indicates that the intratumoral accumulation of nab-paclitaxel is more effective [16, 17]. To date, nab-paclitaxel has been approved for the treatment of metastatic breast cancer [18–20], locally advanced or metastatic non-small cell-lung cancer [21–23], and metastatic pancreatic cancer [24–26]. Not only that, more and more evidences show that nab-paclitaxel is also effective for gastric cancer and peritoneal cancer [27–33].

At present, there are few studies on nab-paclitaxel in the treatment of gastric cancer with peritoneal metastatic cancer by intraperitoneal hyperthermia. In the recent 3 years, nab-paclitaxel is still mainly administered intravenously [34–36], but Markman M and coworkers proved that paclitaxel can have acceptable toxicity and pharmacokinetic advantage for cavity exposure by the intraperitoneal administration [37]. Manzanedo and colleagues used paclitaxel as part of an HIPEC procedure for 1 hour and showed that paclitaxel didn’t negatively affect the prognosis of patients with advanced ovarian cancer [38]. Therefore, we considered whether HIPEC paclitaxel or nab-paclitaxel had the same therapeutic effect in treating patients with gastric cancer. In addition, the stability of hyperthermia drugs plays an important role in efficacy and safety during HIPEC. HIPEC mainly uses the special differences of temperature tolerance between cancer cells and normal tissues to heat the chemotherapy drugs and perfusion fluid to a certain temperature through the intraperitoneal hyperthermic perfusion therapy system, and then continuously circulates and perfuses into the abdominal cavity of patients at constant temperature, so as to remove free cancer cells and small metastatic lesions in the abdominal cavity. Normal tissue cells can continuously tolerate 47 °C for 1 hour under high temperature conditions, while malignant tumor cells can only tolerate 43 °C for 1 hour. 47 °C and 43 °C for 1 hour are regarded as the critical temperatures for irreversible damage of normal tissue cells and malignant tumor cells. However, albumin may be degraded at high temperature. If albumin-bound paclitaxel cannot be guaranteed to be stable under the condition of intraperitoneal hyperthermic perfusion, the therapeutic effect will be affected. Therefore, this topic investigated the stability of albumin-bound paclitaxel drugs at different temperatures, including the determination of the drug content of albumin-bound paclitaxel at 25 °C, 37 °C, 41 °C, 42 °C, and 43 °C, and paclitaxel binding rate, in vitro release rate, determination of human serum albumin content, determination of human serum albumin polymer content, determination of related substances content, etc. This study has laid a foundation for the later study of albumin-bound paclitaxel intraperitoneal hyperthermic perfusion.

## Materials and methods

### Materials

Human albumin reference substance (SLBX9574, purity > 98%) was purchased from Merck. Albumin-bound paclitaxel (B042001249, B042001250, B042001251) was purchased from CSPC Ouyi Pharmaceutical Co., Ltd. (Shijiazhuang, China). 0.9% sodium chloride solution was purchased from Shijiazhuang No.4 Pharmaceutical Co., Ltd. Dipotassium hydrogen phosphate solution (20180625) was purchased from Sinopharm Chemical Reagent Co., Ltd. Paclitaxel reference substance (100382–201,603, purity: 98%) was purchased from National Institutes for Food and Drug Control. Acetonitrile (SHBM9830), dehydrated ethanol (K52258727010)and Methanol was purchased from Merck. Purified water was purchased from Hangzhou Wahaha Co., Ltd. Potassium hydroxide (KOH) (10017018) was purchased from Sinopharm Chemical Reagent Co., Ltd. Glacial acetic acid (20210127) was purchased from Tianjin Kemiou Chemical Reagent Co., Ltd. BAKERBOND Ociadecyl (C18) Disposable Extraction was purchased from J.T.Baker. Cephalomannine reference substance (100926–201,503, HPLC system performance test) was purchased from National Institutes for Food and Drug Control.

### Instruments

High performance liquid chromatography (Waters e2695) was purchased from (Waters, USA), Electronic balance (XS105) was purchased from (Mettler, Switzerland). High performance liquid chromatography (UltiMate 3000) was purchased from (Thermo Electron Corporation, USA). Vortex mixer (IKA MS3) was purchased from (Ikachina, China).

### Determination of human serum albumin content

Preparation of standard curve solution and system suitability test: Placed 65.54 mg human albumin reference substance in a 10 mL measuring flask and diluted to the scale with 0.9% sodium chloride solution, as the control solution (1). Precisely measured 5 mL and 2.5 mL control solution (1) respectively, and placed them in two 10 mL measuring flasks, diluted to the scale with 0.9% sodium chloride solution, as the control solution (2) and (3) (left at room temperature, can be used within 66 h).

Preparation of the test solution: Transferred the albumin-bound paclitaxel with 0.9% sodium chloride solution into a 250 mL measuring flask by subsections and was diluted to the scale with 0.9% sodium chloride solution. Then took 3 mL diluted solution in a water bath at 25 °C, 37 °C, 41 °C, 42 °C and 43 °C for 60 minutes respectively and then determined according to the method.

Analytical condition: Tosohaas TSK G3000 SWXL column (300 × 7.8 mm) was used, column number: 010D04207D, column temperature: 30 °C; detector: UV, wavelength: 232 nm; mobile phase: 0.1 mol/L dipotassium hydrogen phosphate solution (adjusted pH to 7.0 with hydrochloric acid). The flow rate was set at 0.7 mL/min.

### Determination of human serum albumin polymer content

Preparation of test solution: The test solution was taken in the determination of human albumin content section.

Analytical condition: Same condition as the determination of human albumin section, the detection wavelength was set at 280 nm.

### Determination of related substances content

Chromatographic condition: Lichrospher RP C18 analytical column (250 × 4.0 mm), column temperature: 24 °C; Ultraviolet-light detector, wavelength: 232 nm; mobile phase A: methanol-water (1:1), mobile phase B: gradient elution according to the Table 1:

**Table 1:** Gradient elution program

Time(min)	A(%)	B(%)	Flow rate (mL/min)
0	76	24	1.0
50	76	24	1.0
60	0	100	1.0
65	0	100	1.5
70	0	100	1.5
71	76	24	1.0
90	76	24	1.0

Preparation of system suitability solutions A: Placed 20.33 mg paclitaxel reference substance in a 25 mL measuring flask and added 0.01 mol/L potassium hydroxide ethanol solution (added 0.07 g potassium hydroxide in a 100 mL measuring flask and 0.4 mL water to dissolve, diluted to the scale with anhydrous ethanol and mixed well) to 1 mL, shook for 1 minute and then diluted to the scale with 0.1% (V/V) glacial acetic acid methanol solution and shook well. Stored at 4 °C for at least 30 minutes before feeding (stored at 4 °C, can be used within 30 months).

Preparation of system suitability solutions B: Placed 20.33 mg paclitaxel reference substance in a 100 mL measuring flask, dissolved with acetonitrile and diluted to the scale, shook well. Placed 3 mL precisely in a 25 mL measuring flask, diluted to the scale with acetonitrile and shook well. Stored at 4 °C for at least 30 minutes before feeding (stored at 4 °C, can be used within 5 days).

Preparation of system suitability solutions C: Added 0.6 mL system suitability solutions B into a 25 mL measuring flask, diluted to the scale with acetonitrile and shook well. Stored at 4 °C for at least 30 minutes before feeding (stored at 4 °C, can be used within 5 days).

According to elution procedure 1, 10 μL system suitability solution A was injected into the liquid chromatograph and the chromatogram was recorded.

According to elution procedure 2, 10 μL each of system suitability solutions B and C were precisely measured and injected into the liquid chromatograph respectively, and the chromatogram was recorded with 6 consecutive injections.

Formula:

$$\textrm{Self}\ \textrm{control}\ \textrm{impurity}\ \left(\%\right)=\frac{Ai\times RRF}{A}\times 100\%$$

*Ai* is the impurity peak area, *A* is the sum of the peak areas, and *RRF* is relative response factor.

The steps of solid phase extraction: (1) The solid phase extraction column (SPE C18) was activated with 3 mL acetonitrile, followed by two washes with water, 3 mL each time. SPEC18 column was not allowed to run dry. (2) Added 1 mL water into each SPE column, poured the test solution into the SPE column and filled the column with water and left to stand for 10 minutes. (3) Let the test solution slowly pass through the column without running dry, wash three times with water, 3 mL each time, and let the column run dry until the last wash, wiped the receiver down. (4) Added 1 mL acetonitrile to each SPE column and collected the eluate into an HPLC vial. The solution was allowed to store at 4 °C for at least 30 minutes before feeding into the sample.

Preparation of test solution: Took 1 bottle of the product, added 20 mL of 0.9% sodium chloride solution, shook gently to disperse evenly, heated in a water bath at 25 °C, 37 °C, 41 °C, 42 °C and 43 °C for 60 minutes respectively, then placed 305 μL in a 2 mL centrifuge tube, added 600 μL acetonitrile, vortexed for 30 seconds, then extracted by solid phase extraction, the resulting solution was the test solution.

Preparation of blank solution 1: Placed 84 μL human albumin (20%) in a 2 mL centrifuge tube, 600 μL acetonitrile was added precisely, vortexed for 30 seconds, and then the solution was extracted by solid phase extraction. The resulting solution was the blank solution 1.

Preparation of blank solution 2: 600 μL acetonitrile was used for solid phase extraction and the resulting solution was blank solution 2.

Determination method: The test solution and the blank solution 1 and 2 were each measured 10 μL, injected into the liquid chromatograph respectively, eluted according to the gradient procedure 1 in Table 1, recorded the chromatogram, deducted the solvent peak and the impurity peak with impurity content that less than 0.05% according to the corrected peak area normalization method, and calculated the impurity content according to the corrected peak area normalization method.

### In-vitro release rate

Preparation of the test solution: Took 1 bottle of paclitaxel for injection (albumin-binding type), added 20 mL 0.9% sodium chloride solution, shook gently to make it evenly dispersed, took 3 mL and heated it in a water bath at 25 °C, 37 °C, 41 °C, 42 °C and 43 °C for 60 minutes respectively, then took 1 mL of the above solution, put it into a 250 mL measuring bottle. Added simulated human plasma solution (commercially available human albumin preparation mixed with 0.9% sodium chloride solution to obtain simulated plasma solution with a concentration of 5% human albumin) and diluted to the scale. Added 1 mL to a 2 mL round bottom centrifuge tube, prepared 2 portions in parallel, centrifuged at 21,000 g for 60 minutes, put 0.5 mL supernatant into a 5 mL flask, added acetonitrile, sonicate to disperse well, diluted with acetonitrile to the scale and shook well, filtered and take the filtrate as the test solution.

Preparation of the control solution: Placed 10.31 mg paclitaxel reference substance in a 50 mL measuring flask, dissolved and diluted to the scale with acetonitrile, shook well and placed 5 mL in a 50 mL measuring flask, diluted to the scale with acetonitrile, shook well, and 1 mL was measured precisely and placed in a 10 mL measuring flask, diluted to the scale with acetonitrile, shook well. The average of the results of two determinations was taken as the amount of paclitaxel released and recorded as *C*_P2_ (mg/mL). The amount of paclitaxel measured in the determination of paclitaxel content section was taken as the total amount of paclitaxel, and recorded as C_P_ (mg/bottle), and the release rate of paclitaxel was calculated.

Formula:

$$f=\frac{{Wr}\times \textrm{Control}\ \textrm{content}\%}{{Ar}\times {\textrm{dilution}\ \textrm {multiple}}\ {a}}\times 100\%$$

$${\textrm{C}}_{\textrm{p}2}={\textrm{Average}}\ f-{\textrm{value}}\times {Ai}$$

$$\textrm{In}-\textrm{vitro}\ \textrm{release}\ \textrm{rate}\left(\%\right)=\frac{C_{\textrm{P}2}\times 10\times 250\times 20}{C_{\textrm{P}}}\times 100\%$$

*C*_P2_ (mg/mL): the amount of paclitaxel that has been released.

*C*_P_ (mg/bottle): the amount measured in the determination of paclitaxel content section is the amount of total paclitaxel.

Analytical condition: same as determination of paclitaxel content section.

### Paclitaxel binding rate

Preparation of the test solution: Took 1 bottle of the product, added 20 mL 0.9% sodium chloride solution, shook gently to disperse evenly and took 1 mL into a 2 mL round bottom centrifuge tube, centrifuged at 21000 g for 60 minutes, put 0.5 mL supernatant into a 5 mL measuring flask, added an appropriate amount of acetonitrile, and sonicated to disperse evenly, diluted with acetonitrile to the scale and shook well, filtered, and the continuous filtrate was used as the test solution. In addition, took the control solution in the determination of paclitaxel content section, and determined with the same method. Calculated the peak area in external standard method. Took the average of the results of the two test samples as the amount of unconjugated paclitaxel, which was recorded as *C*_P1_ (mg/mL). Took the content measured in the determination of paclitaxel content section as the total amount of paclitaxel, recorded as *C*_P_ (mg/bottle), and calculated the binding rate of paclitaxel.

Formula:

$$f=\frac{{Wr}\times \textrm{Control}\ \textrm{content}\%}{{Ar}\times {\textrm{dilution}\ \textrm{multiple}}\ {a}}\times 100\%$$

$${{C}}_{\textrm{p}1}={\textrm{Average}}\ {f}-{\textrm{value}}\times Ai$$

$$\textrm{Paclitaxel}\ \textrm{binding}\ \textrm{rate}\left(\%\right)=\left(1-{C}_{\textrm{P}1}\times 10\times 20/{C}_{\textrm{P}}\right)\times 100\%$$

*C*_p1_ (mg/mL): the amount of unconjugated paclitaxel.

*C*_P_ (mg/bottle): taking the content measured in the determination of paclitaxel content section as the total amount of paclitaxel.

Analytical condition: same as the analytical condition in determination of paclitaxel content section.

### Determination of paclitaxel content

Preparation of the control solution: Added 10.11 mg and 10.20 mg paclitaxel reference substance in 50 mL volumetric flasks respectively, dissolved and diluted to the scale with acetonitrile, shook well, accurately measured 5 mL, and put them in a 50 mL volumetric flask, and diluted with acetonitrile to the scale, shook well, as a reference solution, put at 4 °C for at least 30 minutes before injection (stored at 4 °C, can be used within 120 hours).

Preparation of the test solution: Took 10 bottles of the product, dispersed them with a small amount of 0.9% sodium chloride solution, and transferred them to a 1000 mL measuring flask with 0.9% sodium chloride solution, diluted to the scale with 0.9% sodium chloride solution, sonicated for 1 minute, measure 5.0 mL precisely, and placed in a 25 mL measuring flask, put in a water bath at 25 °C, 37 °C, 41 °C, 42 °C, 43 °C, respectively, after 60 minutes, diluted to the scale with acetonitrile and sonicated for 5 minutes as the test solution, left at least 30 minutes at 4 °C before sampling (stored at 4 °C, can be used within 28 hours).

Added 20.33 mg paclitaxel reference substance in a 100 mL volumetric flask, dissolved it with acetonitrile and diluted it to the scale, shook well, and used it as the reference substance solution A. Added 12.54 mg cephalomannine reference substance in a 25 mL volumetric flask, dissolved it with acetonitrile and diluted to the scale, shook well, and used it as reference substance solution B. Precisely measure 3 mL reference substance solution A and 1 mL reference substance solution, put them in a 25 mL volumetric flask, diluted to the scale with acetonitrile, and shook well. Left at 4 °C for at least 30 minutes before injection (store at 4 °C, use within 120 hours).

Analytical conditions: Agilent Zorbax SB C18 column (150 × 4.6 mm), column number: USCM053630, column temperature: 30 °C; detector: UV, detection wavelength: 228 nm; mobile phase: acetonitrile-water (1:1).

**Fig. 1:**
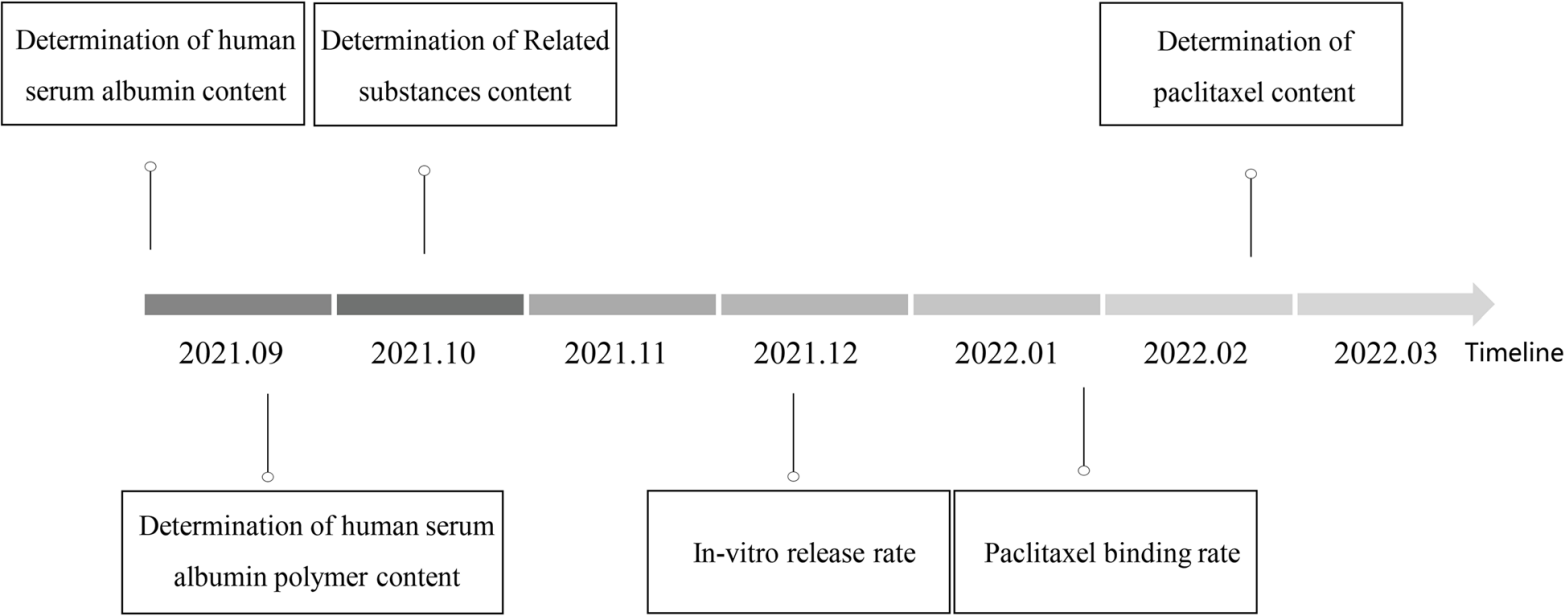
The experimental design with timeline

Above all, we made a schematic representation to intuitively understand the experimental design (Fig. 1).

## Results

### Determination of human serum albumin content

**Fig. 2:**
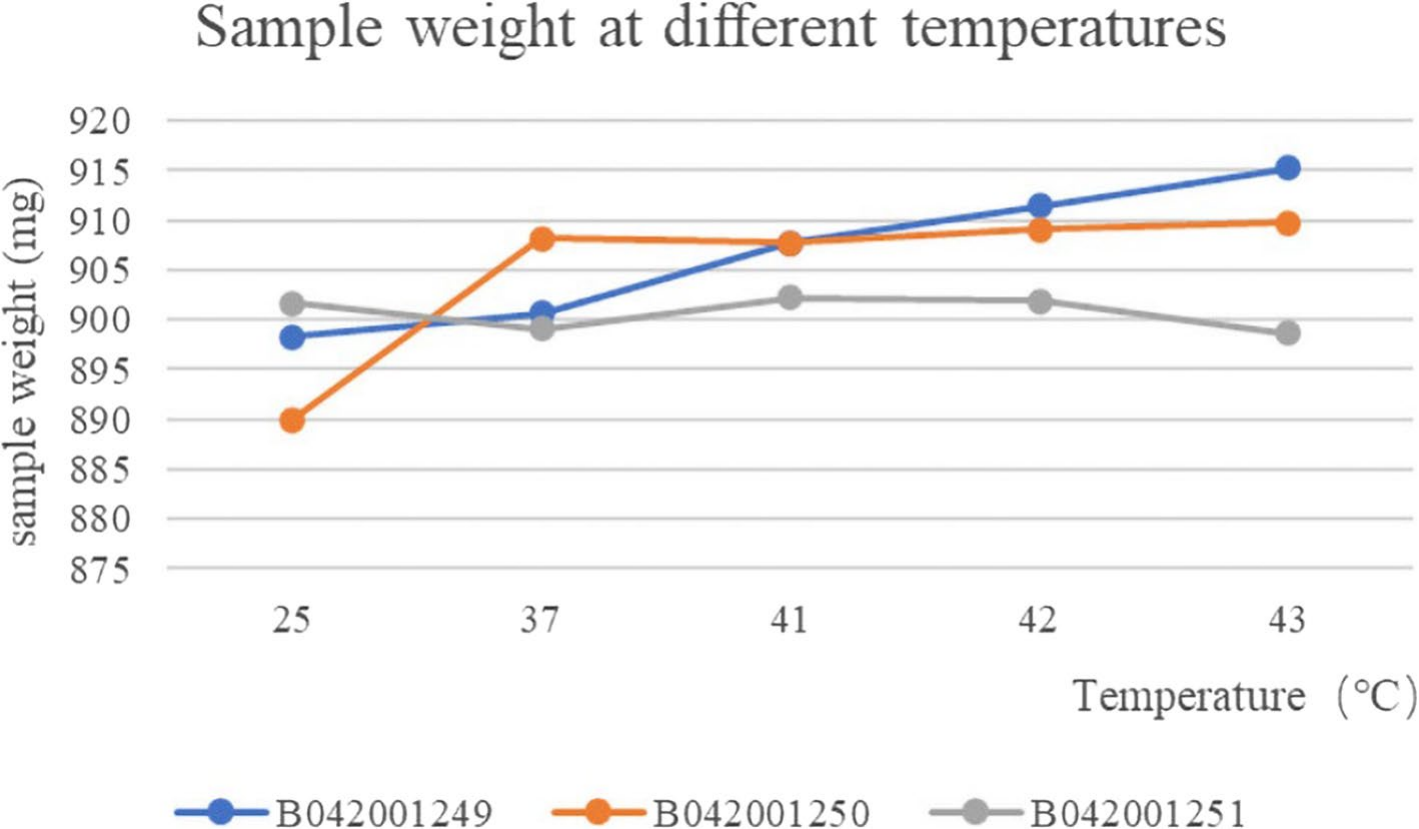
The results of weight changes of three batches of albumin-bound paclitaxel at different temperatures

**Fig. 3:**
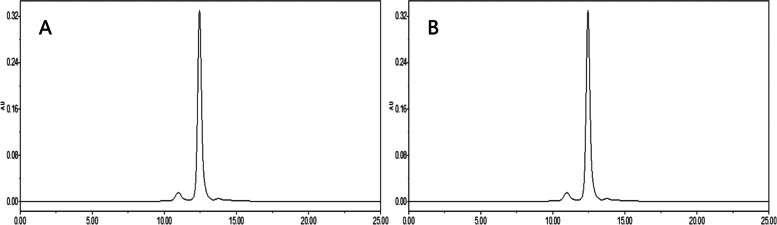
Chromatograms of human albumin reference substance (**A**) and albumin-bound paclitaxel (**B**)

The weight of the three batches of albumin-bound paclitaxel was essentially constant at different temperatures and the RSD values for weight at different temperatures were 0.79, 0.93 and 0.19% respectively, which were stable to temperature (Fig. 2 and Table 2). Chromatograms of dimer (the left peak) and monomer (the right peak) of human albumin reference substance (A) and albumin-bound paclitaxel (B) were shown in Fig. 3.

**Table 2:** The results of content determination of three batches of albumin-bound paclitaxel at different temperatures

Batch number	B042001249	B042001250	B042001251
Temperature(°C)	(mg/bottle)		
25	898.3	890.0	901.7
37	900.7	908.2	899.1
41	907.8	907.7	902.2
42	911.4	909.1	901.9
43	915.3	909.8	898.6

### Determination of human serum albumin polymer content

**Fig. 4:**
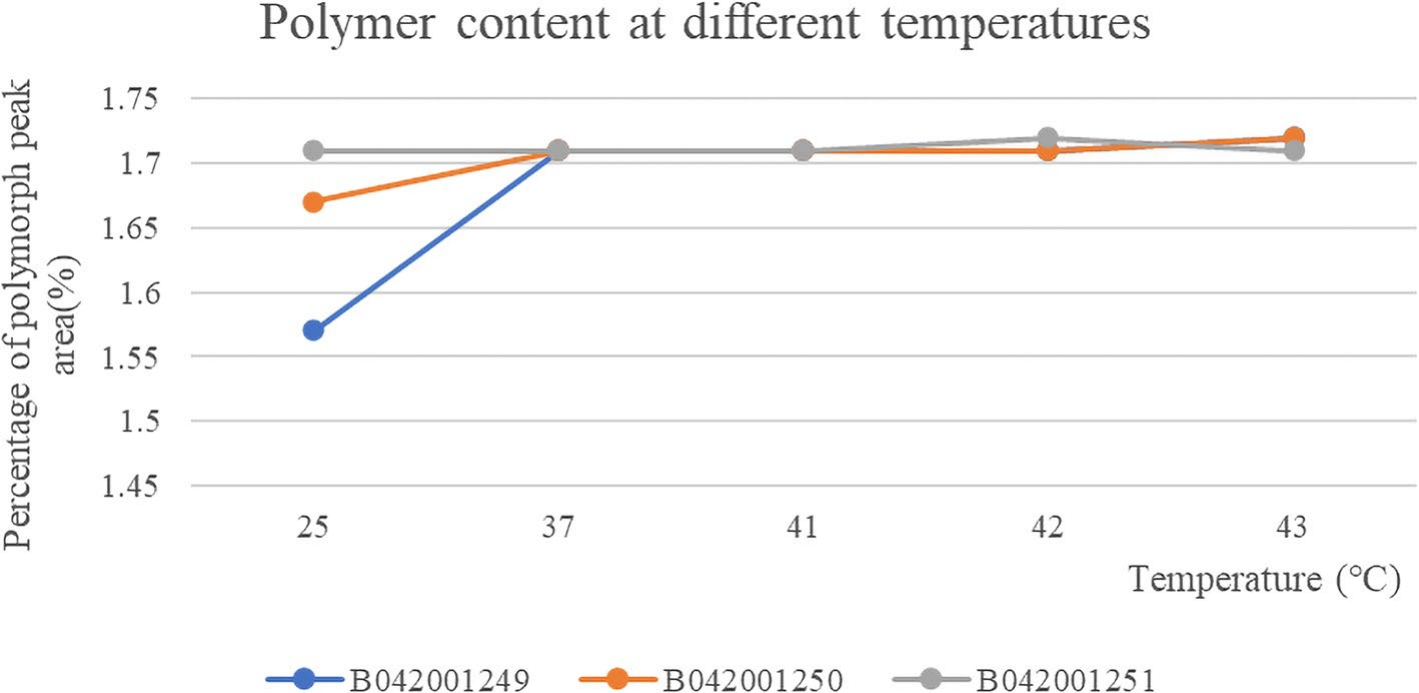
The results of content changes of three batches of polymer of albumin-bound paclitaxel at different temperatures

**Fig. 5:**
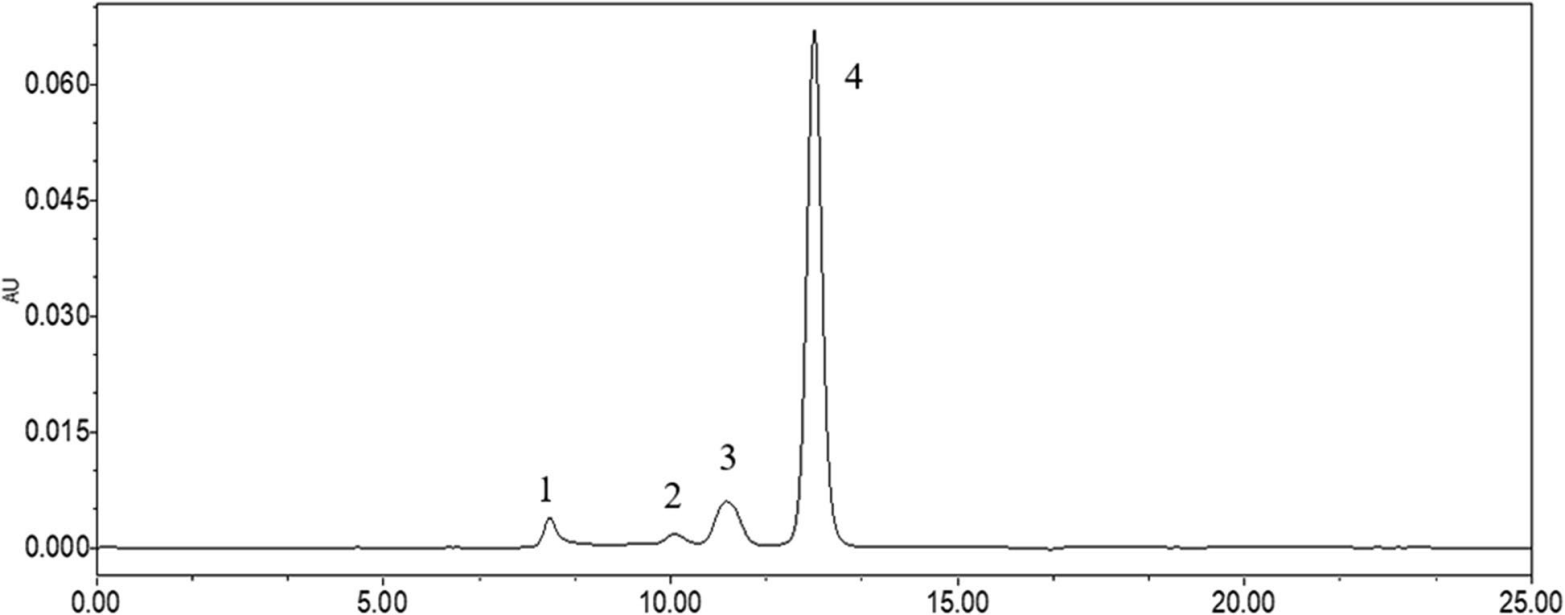
Chromatograms of Polymer (1), Unknown impurities (2), Dimer (3) and Monomer (4)

The content of polymer was basically unchanged in the three batches of samples at different temperatures, and the RSD values of the peak area of polymers at different temperatures were: 3.79, 1.14 and 0.26%, respectively. The polymer did not increase at different temperatures, indicating that paclitaxel (albumin-bound) for injection was stable in saline at different temperatures (Fig. 4 and Table 3). A typical chromatogram of human albumin polymer is shown in Fig. 5.

**Table 3:** The results of polymer peak area of three batches of albumin-bound paclitaxel at different temperatures

Batch number	Temperature (°C)	Polymorph peak area	Total area	Result (%)
B042001249	25	47,721	1,518,743	1.57
	37	52,150	1,525,324	1.71
	41	52,201	1,526,191	1.71
	42	52,203	1,524,651	1.71
	43	52,153	1,518,980	1.72
B042001250	25	51,013	1,525,879	1.67
	37	52,083	1,523,291	1.71
	41	52,070	1,525,968	1.71
	42	52,090	1,520,404	1.71
	43	52,196	1,521,527	1.72
B042001251	25	52,152	1,524,698	1.71
	37	52,080	1,524,160	1.71
	41	52,041	1,523,870	1.71
	42	52,132	1,519,462	1.72
	43	52,003	1,517,695	1.71

### Determination of related substances content

**Fig. 6:**
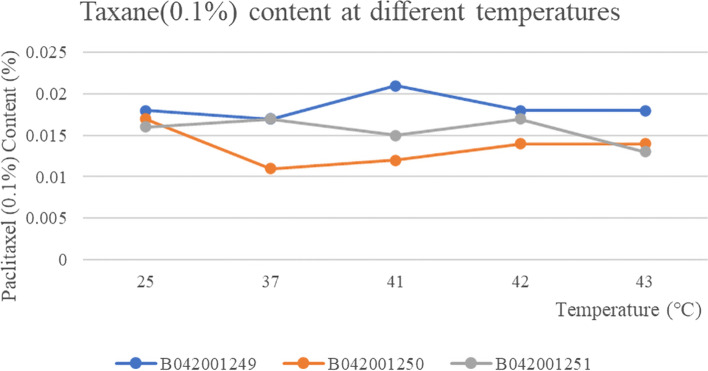
The results of content changes of three batches of taxane of albumin-bound paclitaxel at different temperatures

**Fig. 7:**
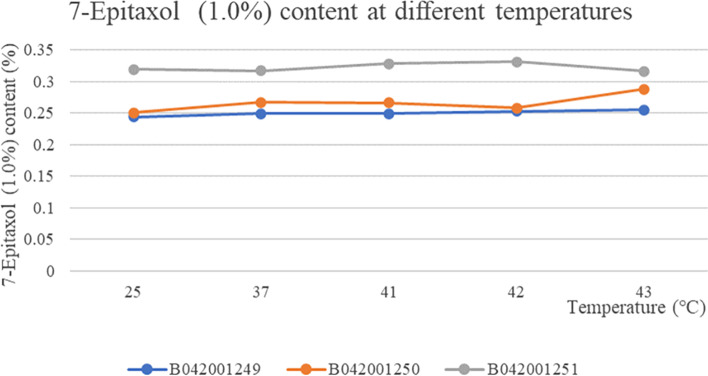
The results of content changes of three batches of 7-Epitaxol of albumin-bound paclitaxel at different temperatures

**Fig. 8:**
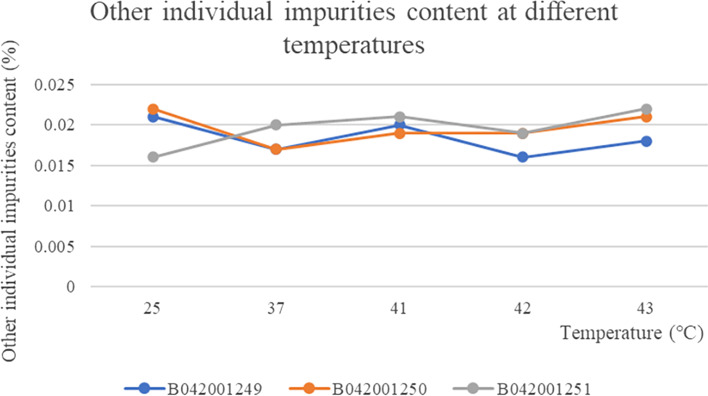
The results of content changes of three batches of other individual impurities of albumin-bound paclitaxel at different temperatures

**Fig. 9:**
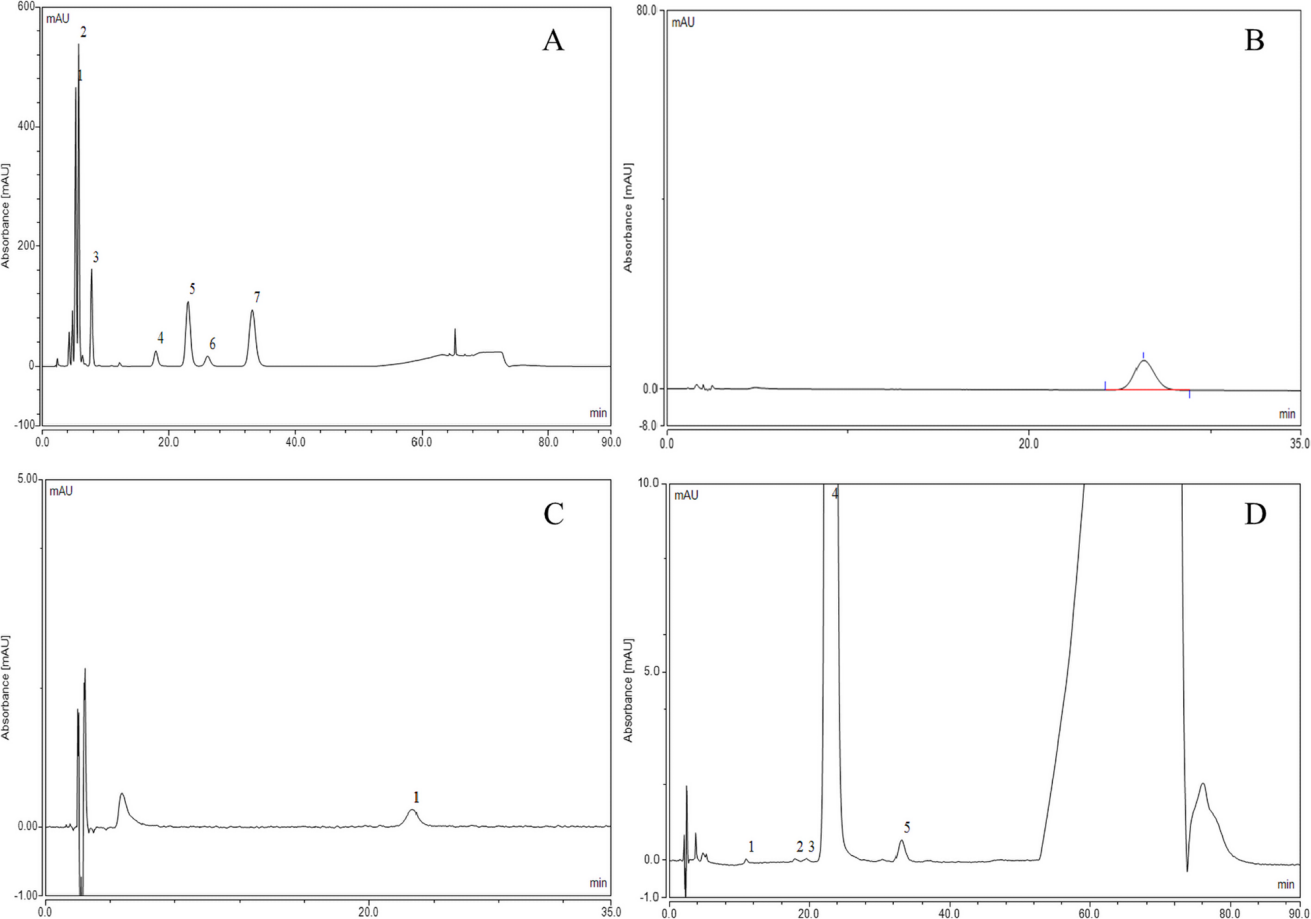
Chromatograms of system suitability solution A (**A**): 2-Baccatin III (2), 1-(2R,3S)-N-benzoyl-3-phenylisoserine methyl ester (3), 5-Paclitaxel (5), 10-Deacetyl-6-7-epipaclitaxel (6), 7–7-Epipaclitaxel (7), system suitability solution B (**B**), system suitability solution C (**C**) and albumin-bound paclitaxel (**D**): 1–20-Taxane (3), 3-Paclitaxel (4), 4–7-Epipaclitaxel (5)

The three batches of taxane of albumin-bound paclitaxel at different temperatures showed no change with the change of temperature and were stable at all temperatures The RSD values for weight at different temperatures were 0.79, 0.93 and 0.19% respectively, which were stable to temperature (Fig. 6). The content of the substance 7-Epitaxol (1%) in the three batches of samples was basically the same at different temperatures and the RSD values for the content of 7-Epitaxol (1.0%) at different temperatures in the three batches of samples were 1.70, 5.23 and 2.12% respectively, so the substance 7-Epitaxol (1%) was stable to temperature (Fig. 7). The other individual maximum impurities at different temperatures were stable at different temperatures with no increase. No new impurities were produced with increasing temperature and the samples were stable at different temperatures (Fig. 8). In addition, Taxane (0.1%) and 7-Epitaxol (1.0%) were detected which was shown in every batch of albumin-bound paclitaxel at different temperatures (Table 4). The chromatogram of related substances can be seen in Fig. 9.

**Table 4:** The relative retention time (*RRT*) and relative response factor (*RRF*) of three batches of related substances of albumin-bound paclitaxel at different temperatures

Batch number: B042001249							
Impurities and degradation products, limits	*RRT*	*RRF*	25 °C	37 °C	41 °C	42 °C	43 °C
(2R,3S)-N-benzoyl-3-phenylisoserine methyl ester (0.1%)	0.21	0.90	Not detected	Not detected	Not detected	Not detected	Not detected
Baccatin III (0.1%)	0.22–0.24	1.00	Not detected	Not detected	Not detected	Not detected	Not detected
(2R,3S)-N-benzoyl-3-phenylisoserine methyl ester (0.1%)	0.24–0.26	0.90	Not detected	Not detected	Not detected	Not detected	Not detected
C3-C11 bridge paclitaxel isomers (0.1%)	0.41	1.00	Not detected	Not detected	Not detected	Not detected	Not detected
10-Deacetylpaclitaxel (0.1%)	0.79–0.80	1.00	Not detected	Not detected	Not detected	Not detected	Not detected
Taxane (0.1%)	0.83–0.84	1.00	0.018	0.017	0.021	0.018	0.018
Cephalomannine (0.4%)	0.89	1.33	Not detected	Not detected	Not detected	Not detected	Not detected
10-Deacetyl-7-epipaclitaxel (0.1%)	1.14	1.00	Not detected	Not detected	Not detected	Not detected	Not detected
7-Epicephalomannine (0.1%)	1.26–1.28	1.31	Not detected	Not detected	Not detected	Not detected	Not detected
7-Epitaxol (1.0%)	1.41–1.45	1.00	0.244	0.249	0.249	0.253	0.255
Paclitaxel C (0.1%)	1.89–2.08	1.80	Not detected	Not detected	Not detected	Not detected	Not detected
N-methyl-paclitaxel C (0.1%)	RT = 57-60 min	1.68	Not detected	Not detected	Not detected	Not detected	Not detected
Other single impurities (0.1%)	–	1.00	0.021	0.017	0.020	0.016	0.018
Total impurities (1.5%)	–	–	0.3	0.3	0.3	0.3	0.3
Batch number: B0420012450							
Impurities and degradation products, limits	*RRT*	*RRF*	25 °C	37 °C	41 °C	42 °C	43 °C
(2R,3S)-N-benzoyl-3-phenylisoserine methyl ester (0.1%)	0.21	0.90	Not detected	Not detected	Not detected	Not detected	Not detected
Baccatin III (0.1%)	0.22–0.24	1.00	Not detected	Not detected	Not detected	Not detected	Not detected
(2R,3S)-N-benzoyl-3-phenylisoserine methyl ester (0.1%)	0.24–0.26	0.90	Not detected	Not detected	Not detected	Not detected	Not detected
C3-C11 bridge paclitaxel isomers (0.1%)	0.41	1.00	Not detected	Not detected	Not detected	Not detected	Not detected
10-Deacetylpaclitaxel (0.1%)	0.79–0.80	1.00	Not detected	Not detected	Not detected	Not detected	Not detected
Taxane (0.1%)	0.83–0.84	1.00	0.017	0.011	0.012	0.014	0.014
Cephalomannine (0.4%)	0.89	1.33	Not detected	Not detected	Not detected	Not detected	Not detected
10-Deacetyl-7-epipaclitaxel (0.1%)	1.14	1.00	Not detected	Not detected	Not detected	Not detected	Not detected
7-Epicephalomannine (0.1%)	1.26–1.28	1.31	Not detected	Not detected	Not detected	Not detected	Not detected
7-Epitaxol (1.0%)	1.41–1.45	1.00	0.251	0.267	0.266	0.258	0.288
Paclitaxel C (0.1%)	1.89–2.08	1.80	Not detected	Not detected	Not detected	Not detected	Not detected
N-methyl-paclitaxel C (0.1%)	RT = 57-60 min	1.68	Not detected	Not detected	Not detected	Not detected	Not detected
Other single impurities (0.1%)	–	1.00	0.022	0.017	0.019	0.019	0.021
Total impurities (1.5%)	–	–	0.3	0.3	0.3	0.3	0.3
Batch number: B042001251							
Impurities and degradation products, limits	*RRT*	*RRF*	25 °C	37 °C	41 °C	42 °C	43 °C
(2R,3S)-N-benzoyl-3-phenylisoserine methyl ester (0.1%)	0.21	0.90	Not detected	Not detected	Not detected	Not detected	Not detected
Baccatin III (0.1%)	0.22–0.24	1.00	Not detected	Not detected	Not detected	Not detected	Not detected
(2R,3S)-N-benzoyl-3-phenylisoserine methyl ester (0.1%)	0.24–0.26	0.90	Not detected	Not detected	Not detected	Not detected	Not detected
C3-C11 bridge paclitaxel isomers (0.1%)	0.41	1.00	Not detected	Not detected	Not detected	Not detected	Not detected
10-Deacetylpaclitaxel (0.1%)	0.79–0.80	1.00	Not detected	Not detected	Not detected	Not detected	Not detected
Taxane (0.1%)	0.83–0.84	1.00	0.016	0.017	0.015	0.017	0.013
Cephalomannine (0.4%)	0.89	1.33	Not detected	Not detected	Not detected	Not detected	Not detected
10-Deacetyl-7-epipaclitaxel (0.1%)	1.14	1.00	Not detected	Not detected	Not detected	Not detected	Not detected
7-Epicephalomannine (0.1%)	1.26–1.28	1.31	Not detected	Not detected	Not detected	Not detected	Not detected
7-Epitaxol (1.0%)	1.41–1.45	1.00	0.319	0.317	0.328	0.331	0.316
Paclitaxel C (0.1%)	1.89–2.08	1.80	Not detected	Not detected	Not detected	Not detected	Not detected
N-methyl-paclitaxel C (0.1%)	RT = 57-60 min	1.68	Not detected	Not detected	Not detected	Not detected	Not detected
Other single impurities (0.1%)	–	1.00	0.016	0.020	0.021	0.019	0.022
Total impurities (1.5%)	–	–	0.4	0.4	0.4	0.4	

*RRT* Relative retention time, *RRF* Relative response factor

### In-vitro release rate

**Fig. 10:**
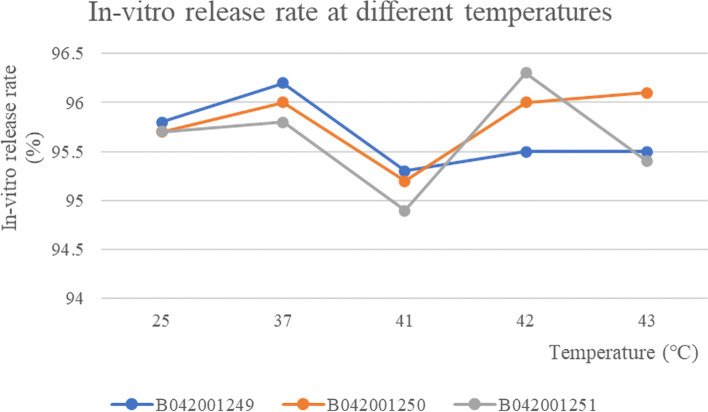
The results of in-vitro release rate of three batches of albumin-bound paclitaxel at different temperatures

**Fig. 11:**
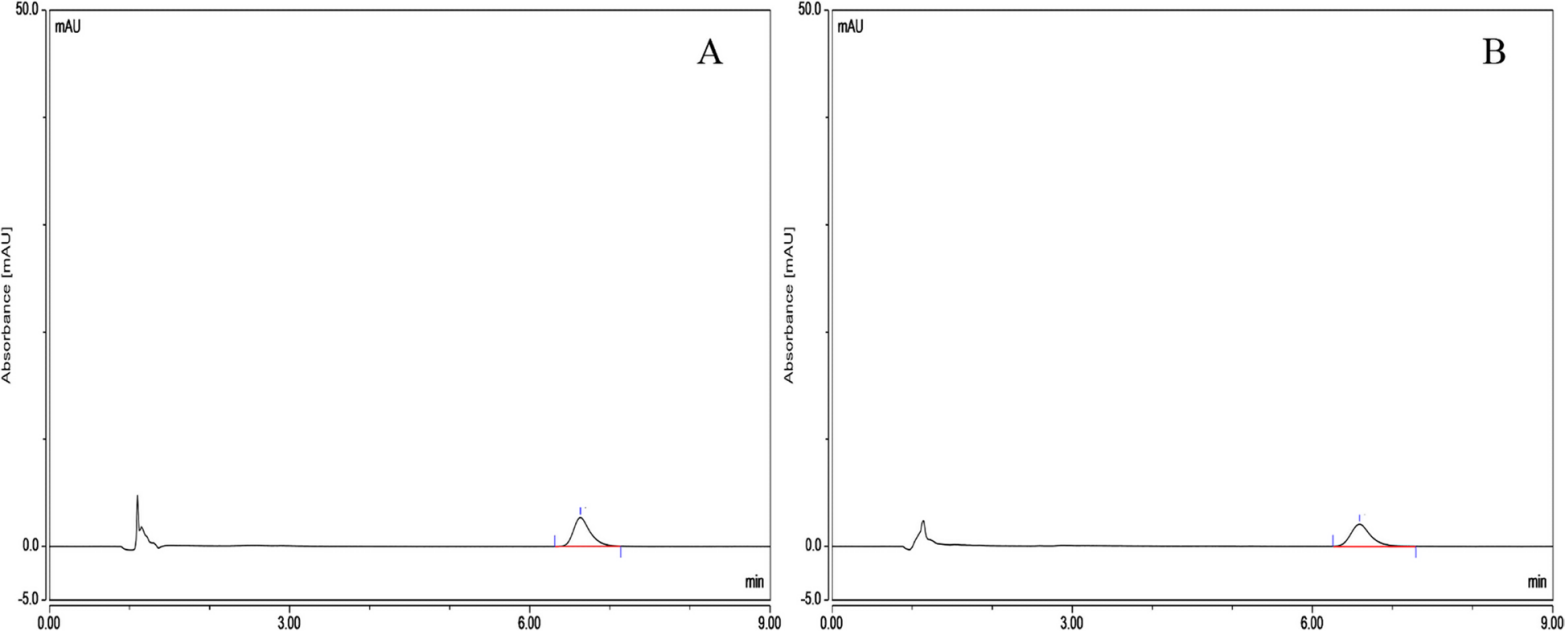
Chromatograms of control solution (**A**) and test solution (**B**)

The In-vitro release rate of three batches of albumin-bound paclitaxel was shown in Table 5 and the RSD values for the in-vitro release rate of the three batches of samples at different temperatures were 0.37, 0.38 and 0.54% respectively, indicating that the temperature had a small effect on the release rate of the drug and the in-vitro release rate were basically the same at different temperatures (Fig. 10). The chromatogram of control solution (A): RT (retention time) was 6.630 minutes and test solution (B): RT (retention time) was 6.590 minutes (Fig. 11).

**Table 5:** The results of peak area, *C*_P_ and release rate *C*_P2_ of three batches of albumin-bound paclitaxel at different temperatures

Batch number	Temperature (°C)	Peak area	*C*_P_ (%)	Release rate *C*_P2_ (%)	Mean value (%)
B042001249	25	0.6286	101.55	96.23	95.8
		0.6224	101.55	95.28	
	37	0.6282	101.55	96.17	96.2
		0.6289	101.55	96.28	
	41	0.6241	101.55	95.54	95.3
		0.6206	101.55	95.01	
	42	0.6217	101.55	95.17	95.5
		0.6254	101.55	95.74	
	43	0.6228	101.55	95.34	95.5
		0.6245	101.55	95.6	
B042001250	25	0.6242	101.36	95.74	95.7
		0.6231	101.36	95.57	
	37	0.6268	101.36	96.13	96.0
		0.625	101.36	95.86	
	41	0.6226	101.36	95.49	95.2
		0.6193	101.36	94.98	
	42	0.6242	101.36	95.74	96.0
		0.627	101.36	96.16	
	43	0.6271	101.36	96.18	96.1
		0.6256	101.36	95.95	
B042001251	25	0.6231	101.54	95.4	95.7
		0.6268	101.54	95.96	
	37	0.6306	101.54	96.55	95.8
		0.6211	101.54	95.09	
	41	0.6197	101.54	94.88	94.9
		0.6202	101.54	94.95	
	42	0.6279	101.54	96.13	96.3
		0.6301	101.54	96.47	
	43	0.6228	101.54	95.35	95.4
		0.6229	101.54	95.37

### Paclitaxel binding rate

**Fig. 12:**
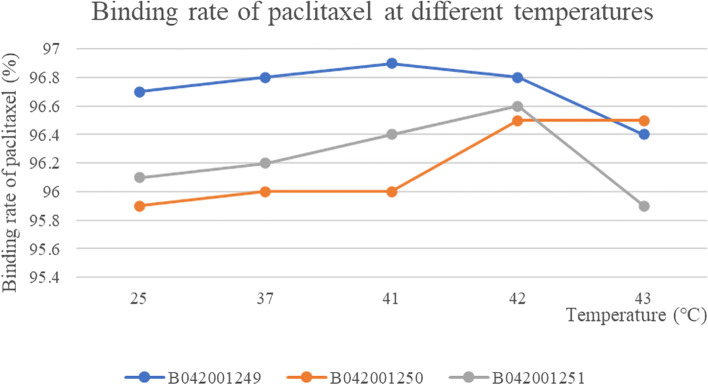
The results of binding rate of three batches of albumin-bound paclitaxel at different temperatures

The RSD value of paclitaxel binding rate at different temperatures for the three batches of samples were: 0.20, 0.31 and 0.28%, respectively. The effect of temperature on the binding rate of paclitaxel was small and stable to temperature (Fig. 12). The peak area, *C*_P_, *C*_P1_ and paclitaxel binding rate of three batches of albumin-bound paclitaxel at different temperatures were shown in Table 6.

**Table 6:** The peak area, *C*_P_, *C*_P1_ and paclitaxel binding rate of three batches of albumin-bound paclitaxel at different temperatures

Batch number	Temperature (°C)	Peak area	*C*_P_	*C*_P1_	Paclitaxel binding rate (%)	Mean value (%)
B042001249	25	6.0256	101.55	0.016708544	96.70	96.7
		6.0806	101.55	0.016861054	96.67	
	37	5.9337	101.55	0.016453712	96.75	96.8
		5.9248	101.55	0.016429033	96.76	
	41	5.685	101.55	0.015764085	96.89	96.9
		5.6808	101.55	0.015752439	96.89	
	42	5.7725	101.55	0.016006716	96.84	96.8
		5.7651	101.55	0.015986196	96.85	
	43	6.5324	101.55	0.018113862	96.43	96.4
		6.54	101.55	0.018134937	96.42	
B042001250	25	7.5575	101.36	0.020956389	95.86	95.9
		7.5572	101.36	0.020955557	95.86	
	37	7.3853	101.36	0.020478891	95.95	96.0
		7.371	101.36	0.020439238	95.96	
	41	7.3002	101.36	0.020242915	96.00	96.0
		7.2888	101.36	0.020211304	96.01	
	42	6.3328	101.36	0.017560386	96.53	96.5
		6.3331	101.36	0.017561218	96.53	
	43	8.2648	101.36	0.02291768	95.47	95.5
		8.2573	101.36	0.022896883	95.48	
B042001251	25	7.2225	101.54	0.020027459	96.05	96.1
		7.2217	101.54	0.02002524	96.05	
	37	6.9538	101.54	0.019282374	96.20	96.2
		6.9525	101.54	0.019278769	96.20	
	41	6.5789	101.54	0.018242804	96.40	96.4
		6.5866	101.54	0.018264155	96.40	
	42	6.2865	101.54	0.017432	96.56	96.6
		6.2864	101.54	0.017431723	96.56	
	43	7.5801	101.54	0.021019057	95.85	95.9
		7.5687	101.54	0.020987446	95.86	

### Determination of paclitaxel content

**Fig. 13:**
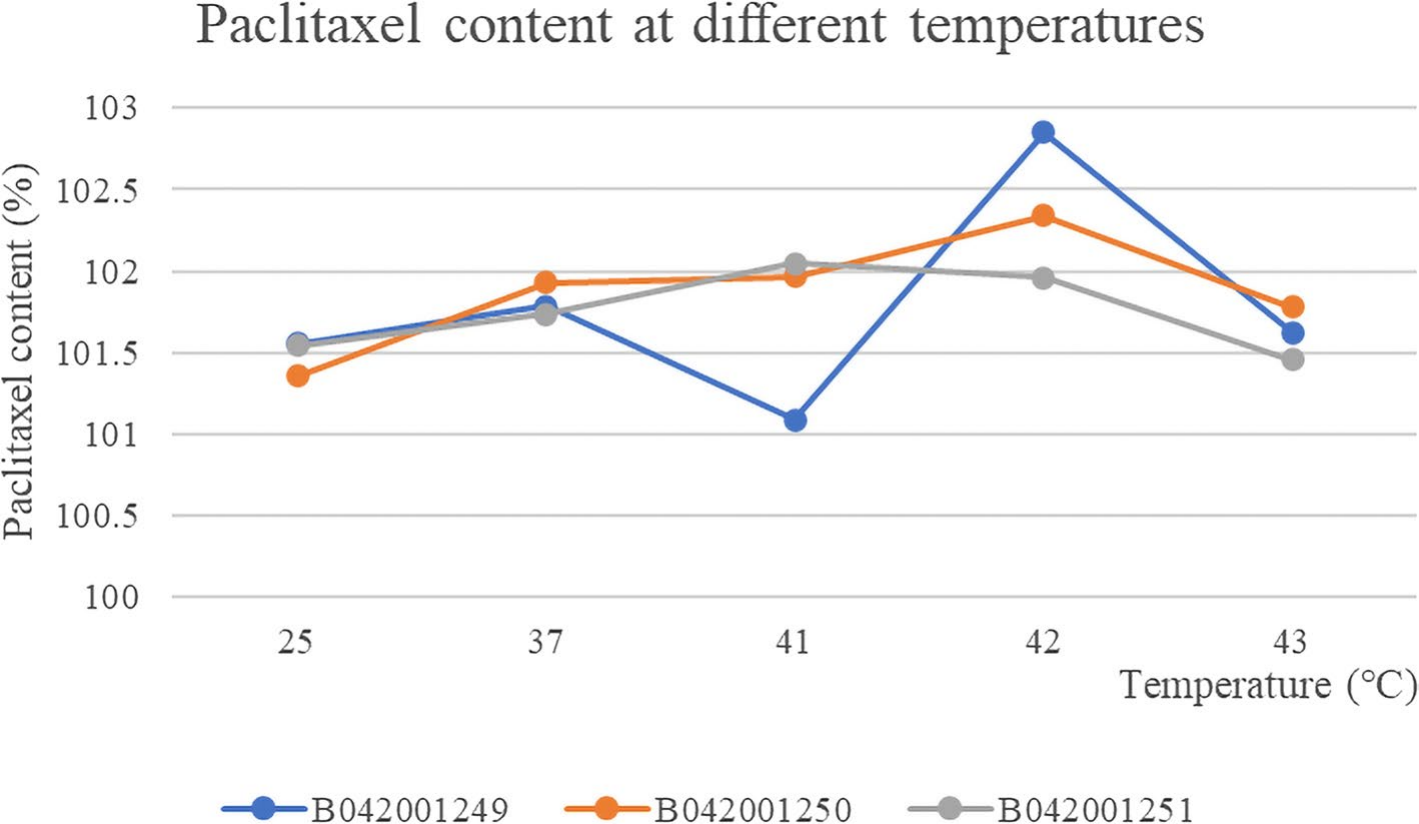
The content results of paclitaxel of three batches of albumin-bound paclitaxel at different temperatures

**Fig. 14:**
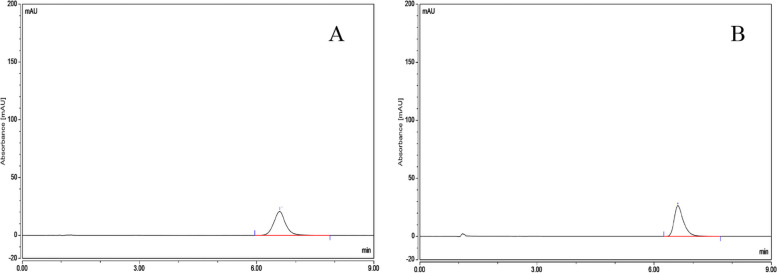
Chromatograms of control solution (**A**) and test solution (**B**)

**Fig. 15:**
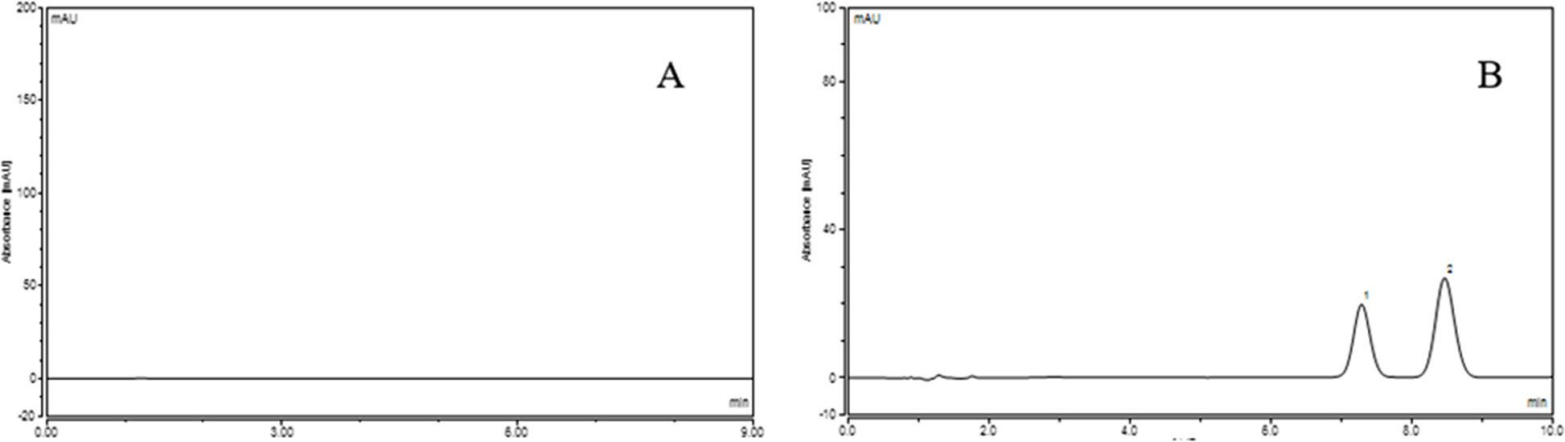
Solvent chromatogram (**A**) and the chromatogram of system suitability solution (**B**): 1-Cephalomannine (1), 2-Paclitaxel (2)

The results (peak area and content) of three batches of albumin-bound paclitaxel in water bath at 25 °C, 37 °C, 41 °C, 42 °C, 43 °C were shown in Table 7, 8, 9, 10 and 11 and the Summary of content results of three batches of albumin-bound paclitaxel in water bath at various temperature conditions was shown in Table 12.The RSDs of paclitaxel content at different temperatures of the three batches of samples were 0.64, 0.35 and 0.26%, respectively. As can be seen from the Fig. 13, the paclitaxel content remained basically the same at different temperatures, indicating that paclitaxel is stable to temperature. The chromatogram of control solution (A): RT (retention time) was 6.588 minutes and test solution (B): RT (retention time) was 6.608 minutes (Fig. 14). Solvent chromatogram (A) and the chromatogram of system suitability solution(B): 1-Cephalomannine (1), 2-Paclitaxel (2) were shown in Fig. 15.

**Table 7:** The results (peak area and content) of three batches of albumin-bound paclitaxel in water bath at 25 °C

Temperature	Batch number	Sample number	Peak area	Content (%)	Mean value (%)
25 °C	B042001249	1	7.3661	102.12	101.554
		2	7.3004	101.21	
		3	7.3212	101.5	
		4	7.3094	101.34	
		5	7.3016	101.23	
		6	7.3864	102.4	
		7	7.3242	101.54	
		8	7.3404	101.77	
		9	7.2974	101.17	
		10	7.304	101.26	
	B042001250	1	7.2889	101.05	101.36
		2	7.1917	99.71	
		3	7.2653	100.73	
		4	7.3685	102.16	
		5	7.4095	102.72	
		6	7.2797	100.93	
		7	7.1981	99.79	
		8	7.2755	100.87	
		9	7.398	102.57	
		10	7.4347	103.07	
	B042001251	1	7.3198	101.48	101.542
		2	7.3458	101.84	
		3	7.2917	101.09	
		4	7.3231	101.53	
		5	7.3709	102.19	
		6	7.3014	101.23	
		7	7.3338	101.68	
		8	7.2636	100.7	
		9	7.3193	101.47	
		10	7.3721	102.21

**Table 8:** The results (peak area and content) of three batches of albumin-bound paclitaxel in water bath at 37 °C

Temperature	Batch number	Sample number	Peak area	Content (%)	Mean value (%)
37 °C	B042001249	1	7.3958	102.54	101.786
		2	7.3504	101.91	
		3	7.3685	102.16	
		4	7.2965	101.16	
		5	7.2936	101.12	
		6	7.4178	102.84	
		7	7.3465	101.85	
		8	7.3573	102	
		9	7.3167	101.44	
		10	7.2736	100.84	
	B042001250	1	7.3061	101.29	101.927
		2	7.3012	101.22	
		3	7.3789	102.3	
		4	7.3939	102.51	
		5	7.3975	102.56	
		6	7.2903	101.07	
		7	7.3314	101.64	
		8	7.3808	102.33	
		9	7.3581	102.01	
		10	7.3818	102.34	
	B042001251	1	7.2706	100.8	101.735
		2	7.3919	102.48	
		3	7.3353	101.7	
		4	7.3306	101.63	
		5	7.3376	101.73	
		6	7.2938	101.12	
		7	7.4079	102.7	
		8	7.3152	101.42	
		9	7.3633	102.08	
		10	7.3352	101.69

**Table 9:** The results (peak area and content) of three batches of albumin-bound paclitaxel in water bath at 41 °C

Temperature	Batch number	Sample number	Peak area	Content (%)	Mean value (%)
41 °C	B042001249	1	7.3442	101.82	101.089
		2	7.2764	100.88	
		3	7.2547	100.58	
		4	7.2691	100.78	
		5	7.272	100.82	
		6	7.3491	101.89	
		7	7.3631	102.08	
		8	7.2501	100.51	
		9	7.2732	100.84	
		10	7.2625	100.69	
	B042001250	1	7.3008	101.22	101.967
		2	7.3877	102.42	
		3	7.3871	102.41	
		4	7.34	101.76	
		5	7.3447	101.83	
		6	7.3331	101.67	
		7	7.3324	101.66	
		8	7.3788	102.3	
		9	7.3763	102.26	
		10	7.3671	102.14	
	B042001251	1	7.3122	101.38	102.049
		2	7.3507	101.91	
		3	7.3686	102.16	
		4	7.3974	102.56	
		5	7.3766	102.27	
		6	7.2984	101.18	
		7	7.3435	101.81	
		8	7.358	102.01	
		9	7.4124	102.77	
		10	7.3886	102.44

**Table 10:** The results (peak area and content) of three batches of albumin-bound paclitaxel in water bath at 42 °C

Temperature	Batch number	Sample number	Peak area	Content (%)	Mean value (%)
42 °C	B042001249	1	7.4453	103.22	102.853
		2	7.3779	102.29	
		3	7.3931	102.5	
		4	7.4412	103.16	
		5	7.4651	103.5	
		6	7.4636	103.48	
		7	7.3539	101.95	
		8	7.3773	102.28	
		9	7.3978	102.56	
		10	7.4719	103.59	
	B042001250	1	7.3539	101.95	102.338
		2	7.3376	101.73	
		3	7.385	102.39	
		4	7.4687	103.55	
		5	7.3915	102.48	
		6	7.3423	101.79	
		7	7.3271	101.58	
		8	7.3617	102.06	
		9	7.481	103.72	
		10	7.3666	102.13	
	B042001251	1	7.2911	101.08	101.96
		2	7.3901	102.46	
		3	7.3333	101.67	
		4	7.3812	102.33	
		5	7.3567	101.99	
		6	7.3112	101.36	
		7	7.3781	102.29	
		8	7.3311	101.64	
		9	7.3826	102.35	
		10	7.388	102.43

**Table 11:** The results (peak area and content) of three batches of albumin-bound paclitaxel in water bath at 43 °C

Temperature	Batch number	Sample number	Peak area	Content (%)	Mean value (%)
43 °C	B042001249	1	7.3777	102.28	101.627
		2	7.3343	101.68	
		3	7.3121	101.37	
		4	7.3222	101.51	
		5	7.3126	101.38	
		6	7.3758	102.26	
		7	7.3535	101.95	
		8	7.3131	101.39	
		9	7.3212	101.5	
		10	7.2816	100.95	
	B042001250	1	7.2478	100.48	101.779
		2	7.31	101.35	
		3	7.3119	101.37	
		4	7.4031	102.64	
		5	7.4141	102.79	
		6	7.2651	100.72	
		7	7.3212	101.5	
		8	7.3271	101.58	
		9	7.392	102.48	
		10	7.4208	102.88	
	B042001251	1	7.3407	101.77	101.457
		2	7.3106	101.35	
		3	7.3262	101.57	
		4	7.3377	101.73	
		5	7.3047	101.27	
		6	7.3478	101.87	
		7	7.2745	100.85	
		8	7.3221	101.51	
		9	7.3416	101.78	
		10	7.2754	100.87

**Table 12:** The Summary of content results of three batches of albumin-bound paclitaxel in water bath at 25 °C, 37 °C, 41 °C, 42 °C, 43 °C

Batch number	B042001249	B042001250	B042001251
Temperature	Content (%)		
25 °C	101.55	101.36	101.54
37 °C	101.79	101.93	101.74
41 °C	101.09	101.97	102.05
42 °C	102.85	101.96	101.63
43 °C	101.63	101.78	101.46

## Discussion

Gastric cancer is a major disease in modern time and now it is the fifth most common cancer [39]. In the past few decades, surgical resection has played a crucial role in the treatment of GC [40]. Currently, there are relatively few studies on HIPEC in gastric cancer patients, but the number of related studies is gradually increasing.

Considering that there is a lack of basic research on albumin-bound paclitaxel in intraperitoneal hyperthermia chemotherapy and the therapy requires a temperature of 43 °C, this experiment was designed to investigate the stability of nab-paclitaxel at this temperature and at 25 °C, 37 °C, 41 °C, 42 °C and 43 °C. In the process of hyperthermic intraperitoneal chemotherapy, adequate physicochemical stability is critical to ensure safety and efficacy on clinically relevant conditions. Therefore, we mainly focus on the content of human albumin, polymer content, related substance content, in-vitro release rate, paclitaxel binding rate, paclitaxel content, changes in substance content to determine whether there were significant differences in the stability of the drugs at different temperatures. The results that we described show that albumin-bound paclitaxel is relatively stable to different temperatures.

Compared with intravenous injection, hyperthermic intraperitoneal chemotherapy increases the antitumor effect of hyperthermia and the synergistic effect of chemotherapy drugs on the basis of intraperitoneal infusion chemotherapy. At the same time, hyperthermia can also increase the penetration of the drug in the tissue, and the systemic adverse reactions are small, which can improve the quality of life of patients with abdominal metastasis. However, there are few studies on the thermal stability of antitumor drugs under the temperature conditions required for intraperitoneal thermal perfusion.

From the above discussion, although we have proved that nab-paclitaxel is stable at different temperatures when dissolved in saline, this experiment lacks the investigation of the effect of concentration on the stability of nab-paclitaxel at different temperatures and the maximum time to maintain stability. Moreover, The entire experiment was conducted in vitro, ignoring the situation of the stability of nab-paclitaxel in vivo and its metabolism, so it’s necessary to conduct animal experiments under the premise of further refinement of the experimental procedure to lay a good foundation for future clinical application.

## Conclusion

Three batches of albumin-bound paclitaxel were dissolved in saline at different temperatures (25 °C, 37 °C, 41 °C, 42 °C and 43 °C) to examine that the changes of human blood albumin content, polymer content, in-vitro release rate, paclitaxel binding rate and paclitaxel content were stable to the several temperatures. With the change in temperature, Taxane (0.1%) and other impurities in the determination of related substance content fluctuated comparatively widely. Aside from Taxane (0.1%), only 7-Epitaxol (1%) was detected. Overall, the drug is relatively stable to temperature.

## Data Availability

Data analyzed and used for this manuscript are available within the manuscript.
